# Supplementary material for: Detection of quantum-vacuum field correlations outside the light cone
Source: Nat Commun. 2022 Jun 13;13:3383. doi: 10.1038/s41467-022-31081-1 (PMC9192708; doi:10.1038/s41467-022-31081-1)
Supplement: Supplementary file 1 — Supplementary Information [file 41467_2022_31081_MOESM1_ESM.pdf]

Supplementary Information for 'Detection of quantum-vacuum field correlations outside the light cone'

Francesca Fabiana Settembrini,[*,1] Frieder Lindel,[*,2] Alexa Marina Herter,[1] Stefan Yoshi Buhmann,[3] and Jérôme Faist[*,1]

[1]*ETH Zurich, Institute of Quantum Electronics, Auguste-Piccard-Hof 1, 8093 Zurich, Switzerland*

[2]*Physikalisches Institut, Albert-Ludwigs-Universität Freiburg, Hermann-Herder-Straße 3, 79104 Freiburg, Germany*

[3]*Institut für Physik, Universität Kassel, Heinrich-Plett-Straße 40, 34132 Kassel, Germany*

* E-mail: fsettemb@phys.ethz.ch; frieder.lindel@physik.uni-freiburg.de; jfaist@ethz.ch

Supplementary Note 1: Vacuum correlation function

The quantized electric field operator in general absorptive and dispersive optical environments described by the permittivity $\epsilon(\Omega)$ can be obtained using the framework of macroscopic quantum electrodynamics (QED)[1]. We start with expanding the field in its frequency components

$$\hat{\mathbf{E}}(\mathbf{r},t) = \int_0^\infty \mathrm{d}\Omega \, \mathrm{e}^{-\mathrm{i}\Omega t} \hat{\mathbf{E}}(\mathbf{r},\Omega) + \mathrm{H.c.} \tag{1}$$

Either by canonically quantizing classical macroscopic quantum electrodynamics[2] or by using the fluctuation-dissipation theorem[3] one can show, that the ground-state two-point correlation function of $\hat{\mathbf{E}}(\mathbf{r},\Omega)$ is given by[1]

$$\langle\hat{\mathbf{E}}_{\rm vac}(\mathbf{r},\Omega)\hat{\mathbf{E}}^\dagger_{\rm vac}(\mathbf{r}',\Omega')\rangle = \frac{\hbar\Omega^2}{c^2\varepsilon_0\pi}\delta(\Omega-\Omega'){\rm Im}\mathsf{D}(\mathbf{r},\mathbf{r}',\Omega). \tag{2}$$

Here D is the Green's tensor of the vector Helmholtz equation defined via

$$\left(\nabla\times\nabla\times-\frac{\Omega^2}{c^2}\varepsilon(\Omega)\right)\mathsf{D}(\mathbf{r},\mathbf{r}',\Omega) = \boldsymbol{\delta}(\mathbf{r}-\mathbf{r}'), \tag{3}$$

with the boundary condition $\mathsf{D}(\mathbf{r},\mathbf{r}',\Omega)\to 0$ for $|\mathbf{r}-\mathbf{r}'|\to\infty$. We are only interested in the xx component of the Green's tensor which is most conveniently expressed as[1]

$$\mathsf{D}_{xx}(\mathbf{r},\mathbf{r}',\Omega) = -\frac{1}{3k^2}\delta(\boldsymbol{\rho}) - \frac{{\rm e}^{{\rm i}k\rho}}{4\pi k^2\rho^3}\left\{\left[1-{\rm i}k\rho-(k\rho)^2\right]-\frac{\rho_x^2}{\rho^2}\left[3-3{\rm i}k\rho-(k\rho)^2\right]\right\}, \tag{4}$$

with $\boldsymbol{\rho}=\mathbf{r}-\mathbf{r}'$, $\rho=|\mathbf{r}-\mathbf{r}'|$ and $k=n(\Omega)\Omega/c$. In the retarded limit, i.e. for $\rho k\gg 1$, which will be used in Supplementary Note 2 B below we find

$$\mathsf{D}_{xx}(\mathbf{r},\mathbf{r}',\Omega) = \left(1-\frac{\rho_x^2}{\rho^2}\right)\frac{{\rm e}^{{\rm i}k\rho}}{4\pi\rho}. \tag{5}$$

This can be seen by expanding the exponentials in Eq. (4) in powers of ρk. Expressing the correlator of the electric field in time domain in terms of the correlator in frequency space we find

$$\begin{aligned}\{\hat{E}_x(\mathbf{r},t),\hat{E}_x(\mathbf{r}',t')\} &= \int_0^\infty{\rm d}\Omega\int_0^\infty{\rm d}\Omega'{\rm e}^{-{\rm i}(\Omega t-\Omega' t')}\langle\hat{E}_x(\mathbf{r},\Omega)\hat{E}_x^\dagger(\mathbf{r}',\Omega')\rangle \\ &\qquad + {\rm e}^{{\rm i}(\Omega t-\Omega' t')}\langle\hat{E}_x(\mathbf{r}',\Omega')\hat{E}_x^\dagger(\mathbf{r},\Omega)\rangle \qquad (6)\\ &= \int_0^\infty{\rm d}\Omega\int_0^\infty{\rm d}\Omega'\langle\hat{E}_x(\mathbf{r},\Omega)\hat{E}_x^\dagger(\mathbf{r}',\Omega')\rangle({\rm e}^{-{\rm i}(\Omega t-\Omega' t')}+{\rm e}^{{\rm i}(\Omega' t-\Omega t')}) \qquad (7)\\ &= \frac{2\hbar\mu_0}{\pi}\int_0^\infty{\rm d}\Omega\,\Omega^2\cos(\Omega\tau){\rm Im}[\mathsf{D}_{xx}(\mathbf{r},\mathbf{r}',\Omega)]. \qquad (8)\end{aligned}$$

Using Eq. (4) and neglecting absorption such that k is real, this can be further simplified to yield

$$\{\hat{E}_x(\mathbf{r},t),\hat{E}_x(\mathbf{r}',t')\} = \frac{\hbar\mu_0}{2\pi^2\rho^3}\int_0^\infty \mathrm{d}\Omega\,\frac{\Omega^2}{k^2}\cos(\Omega\tau)$$
$$\times\left\{\left[\sin(k\rho)(k^2\rho^2-1)+k\rho\cos(k\rho)\right]-\frac{\rho_x^2}{\rho^2}\left[\sin(k\rho)(k^2\rho^2-3)+3k\rho\cos(k\rho)\right]\right\}. \quad (9)$$

Assuming $\rho_x/\rho \ll 1$ and $\rho k \gg 1$ one finds

$$\{\hat{E}_x(\mathbf{r},t),\hat{E}_x(\mathbf{r}',t')\} = \frac{\hbar\mu_0}{2\pi^2\rho}\int_0^\infty \mathrm{d}\Omega\,\Omega^2\cos(\Omega\tau)\sin(k\rho). \quad (10)$$

Supplementary Note 2: Electro-optic sampling in space and time

A. Full calculation

In this section we calculate the EOS signal allowing for the full, medium-assisted quantum vacuum inside the nonlinear crystal as predicted by macroscopic QED with the only approximation that absorption effects are neglected. This is a reasonable assumption in the accessed THz frequency range, see Supplementary Refs.[4,5] for a thorough discussion of absorption effects in EOS.

The electro-optic sampling signal accounting for dispersion inside the nonlinear crystal and the full medium-assisted quantum vacuum, including counter rotating terms, has been derived using macroscopic quantum electrodynamics and it reads[4,5]

$$g^{(1)}(\delta t,\delta r_\perp) = \int_{V_\mathrm{C}}\mathrm{d}^3r\int_{V_\mathrm{C}}\mathrm{d}^3r'\int_0^\infty\mathrm{d}\Omega\int_0^\infty\mathrm{d}\Omega' F(\mathbf{r},\mathbf{r}',\Omega,\Omega')\langle\hat{E}_x(\mathbf{r},\Omega)\hat{E}_x^\dagger(\mathbf{r}',\Omega')\rangle. \quad (11)$$

Here, V_C is the volume of the nonlinear crystal and $F(\mathbf{r},\mathbf{r}',\Omega,\Omega')$ is the filter function in frequency domain given in the laser-paraxial approximation introduced in Supplementary Ref.[5] by

$$F(\mathbf{r},\mathbf{r}',\Omega,\Omega') = 2\left(\frac{2\chi^{(2)}N\omega_p}{\pi c\epsilon_0 w^2 n(\omega_c)}\right)^2\Gamma(\Omega)\Gamma(\Omega')\mathrm{e}^{-\mathrm{i}n_g(\Omega y-\Omega' y')/c}$$
$$\times\left[\mathrm{e}^{-2\left[\mathbf{r}_\perp^2+(\mathbf{r}'_\perp+\delta\mathbf{r}_\perp)^2\right]/w^2}\mathrm{e}^{\mathrm{i}\Omega'\delta t}+\mathrm{e}^{-2\left[\mathbf{r}'^2_\perp+(\mathbf{r}_\perp+\delta\mathbf{r}_\perp)^2\right]/w^2}\mathrm{e}^{-\mathrm{i}\Omega\delta t}\right]. \quad (12)$$

$\chi^{(2)}$ is the nonlinear susceptibility of the crystal, c is the speed of light in vacuum, ϵ_0 is the vacuum permittivity, $n(\omega_c)$ is the refractive index at the central frequency of the laser pulses ω_c, N is the number of detected photons, ω_p is the averaged detected frequency and $\Gamma(\Omega) = \mathrm{e}^{-\Omega^2\tau_\sigma^2/8}$ is the spectral autocorrelation function. Note that τ_σ is connected to the full width half maximum (FWHM) of the laser pulses τ_p via $\tau_\sigma = \tau_p/\sqrt{2\ln(2)}$. τ_p has been determined experimentally and is given by $\tau_p = 195\,\mathrm{fs}$, see Supplementary Note 2 D for details. Furthermore, $\perp$ denotes the plane perpendicular to the laser propagation direction such that $\mathbf{r}_\perp = (x, 0, z)^T$.

Equation (11) can be transformed to the time domain by inserting the inverse Fourier transform of Eq. (1), i.e.

$$\hat{\mathbf{E}}(\mathbf{r}, \Omega) = \frac{1}{2\pi}\int_{-\infty}^{\infty} \mathrm{d}t\, \mathrm{e}^{\mathrm{i}\Omega t}\hat{\mathbf{E}}(\mathbf{r}, t). \tag{13}$$

This leads, after some algebra, to Eq. (2) of the main text which we repeat here

$$G^{(1)}(\delta t, \delta r_\perp) = \int \mathrm{d}t \int_{V_C}\mathrm{d}^3r \int \mathrm{d}t' \int_{V_C}\mathrm{d}^3r' L_1(\mathbf{r}, t) L_2(\mathbf{r}', t')\langle\{\hat{E}_x(\mathbf{r}, t), \hat{E}_x(\mathbf{r}', t')\}\rangle. \tag{14}$$

Note, that $\int \mathrm{d}t \equiv \int_{-\infty}^{\infty} \mathrm{d}t$ and that we normalize the anti-commutator such that

$$\{\hat{E}_x(\mathbf{r}, t), \hat{E}_x(\mathbf{r}', t')\} = \frac{1}{2}[\hat{E}_x(\mathbf{r}, t)\hat{E}_x(\mathbf{r}', t') + \hat{E}_x(\mathbf{r}', t')\hat{E}_x(\mathbf{r}, t)]. \tag{15}$$

In Eq. (14) we have defined the EOS sampling signal $G^{(1)}(\delta t, \delta r_\perp) = g^{(1)}(\delta t, \delta r_\perp)/C$ normalized by the detector efficiency C which reads

$$\sqrt{C} = \frac{2L\chi^{(2)}N\omega_p}{\epsilon_0 c n(\omega_c)}. \tag{16}$$

Furthermore, the space-time volumes of the laser pulses as functions of position and time are given by

$$L_1(\mathbf{r}, t) = \left(\frac{2}{\pi}\right)^{3/2} \frac{1}{\tau_\sigma w^2 L} \mathrm{e}^{-2(n_g \frac{y}{c} - t)^2/\tau_\sigma^2} \mathrm{e}^{-2r_\perp^2/w^2} \tag{17}$$

$$L_2(\mathbf{r}', t') = L_1(\mathbf{r}' + \delta r_\perp \mathbf{e}_x, t' + \delta t). \tag{18}$$

Here, $\mathbf{e}_x$ is the unit vector in x direction. Note, that in Eq. (14) one integrates over the arguments of $L_1(\mathbf{r}, t)$ and $L_2(\mathbf{r}', t')$ separately such that one still finds non-zero contributions

to $G^{(1)}$ even if the overlap between L_1 and L_2 is exactly zero.

To be able to discuss the contributions form correlations between causally and non-causally connected space-time regions in Supplementary Note 2 C we substitute relative coordinates $\boldsymbol{\rho} = \mathbf{r} - \mathbf{r}'$ and $\tau = t - t'$. This enables one to distinguish causal and non-causal space-time points via $\rho < |\tau| c_n$ and $\rho > |\tau| c_n$, respectively. To simplify the resulting expression we introduce spherical coordinates:

$$\int_{-\infty}^{\infty} \mathrm{d}t \int_{V_C} \mathrm{d}^3 r = \int_{-\infty}^{\infty} \mathrm{d}\tau \int \mathrm{d}^2 \boldsymbol{\rho}_\perp \int_{-L/2-y'}^{L/2-y'} \mathrm{d}\rho_y = \int_{-\infty}^{\infty} \mathrm{d}\tau \int\limits_0^{\infty} \mathrm{d}\rho\, \rho^2 \int\limits_0^{2\pi} \mathrm{d}\phi \int\limits_{-\min[\frac{L/2+y'}{\rho},1]}^{\min[\frac{L/2-y'}{\rho},1]} \mathrm{d}\cos(\theta). \tag{19}$$

Inserting this in Eq. (14) and using that $S(\boldsymbol{\rho}, \tau) \equiv \langle \{ \hat{E}_x(\mathbf{r}, t), \hat{E}_x(\mathbf{r}', t') \} \rangle$ only depends on $\boldsymbol{\rho}$ and τ such that the $\mathbf{r}'_\perp$ and t' integrals can be carried out, we get

$$G^{(1)}_{\mathrm{eo}}(\delta t, \delta r_\perp) = \frac{1}{2\pi^{3/2} \tau_\sigma w^2 L^2} \int_{-\infty}^{\infty} \mathrm{d}\tau \int_0^{\infty} \mathrm{d}\rho\, \rho^2 \int_{-L/2}^{L/2} \mathrm{d}y' \int_0^{2\pi} \mathrm{d}\phi \int_{-\min[\frac{L/2+y'}{\rho},1]}^{\min[\frac{L/2-y'}{\rho},1]} \mathrm{d}\cos(\theta)$$
$$\times S(\boldsymbol{\rho}, \tau) \mathrm{e}^{-\frac{\left(\frac{n_g \rho_y}{c} + \delta t - \tau\right)^2}{\tau_\sigma^2} - \frac{(\boldsymbol{\rho}_\perp - \delta r_\perp \mathbf{e}_x)^2}{w^2}}. \tag{20}$$

Realizing that the integrand is independent of y', we can integrate by parts and make use of the Leibniz integral rule to find

$$G^{(1)}_{\mathrm{eo}}(\delta t, \delta r_\perp) = \frac{1}{2\pi^{3/2} \tau_\sigma w^2 L} \int_{-\infty}^{\infty} \mathrm{d}\tau \int\limits_0^{\infty} \mathrm{d}\rho\, \rho^2 \int\limits_0^{2\pi} \mathrm{d}\phi \int\limits_{-\min[\frac{L}{\rho},1]}^{\min[\frac{L}{\rho},1]} \mathrm{d}\cos(\theta)$$
$$\times \left(1 - \frac{\rho}{L} |\cos(\theta)|\right) S(\boldsymbol{\rho}, \tau) \mathrm{e}^{-\frac{\left(\frac{n_g \rho_y}{c} + \delta t - \tau\right)^2}{\tau_\sigma^2} - \frac{(\boldsymbol{\rho}_\perp - \delta r_\perp \mathbf{e}_x)^2}{w^2}}. \tag{21}$$

Inserting the two point correlation function in Eq. (8) and carrying out the ϕ integral one

obtains

$$G^{(1)}_{\mathrm{eo}}(\delta t, \delta r_\perp) = \frac{\hbar}{\epsilon_0 c^2 \pi} \int\limits_{-\infty}^{\infty} \mathrm{d}\tau \int\limits_{0}^{\infty} \mathrm{d}\rho \int\limits_{0}^{\infty} \mathrm{d}\Omega \, \frac{\Omega^2}{k^2 \rho} \cos(\Omega\tau)$$

$$\times \left\{ F_1(\rho,\tau) \left[\sin(k\rho)(k^2\rho^2 - 1) + k\rho\cos(k\rho) \right] - F_2(\rho,\tau) \left[\sin(k\rho)(k^2\rho^2 - 3) + 3k\rho\cos(k\rho) \right] \right\}, \tag{22}$$

with

$$F_1(\rho,\tau) = \frac{1}{2\tau_\sigma w^2 L \pi^{3/2}} \int\limits_{-\min[\frac{L}{\rho},1]}^{\min[\frac{L}{\rho},1]} \mathrm{d}\cos(\theta) \left(1 - \frac{\rho}{L} |\cos(\theta)| \right) J_0 \left(\frac{2\rho\delta r_\perp \sin(\theta)}{w^2} \right)$$
$$\times \, \mathrm{e}^{-\frac{\delta r_\perp^2 + \rho^2 \sin^2(\theta)}{w^2}} \mathrm{e}^{-\frac{\left(\frac{n_g \rho y}{c} + \delta t - \tau\right)^2}{\tau_\sigma^2}}, \tag{23}$$

$$F_2(\rho,\tau) = \frac{1}{2\tau_\sigma w^2 L \pi^{3/2}} \int\limits_{-\min[\frac{L}{\rho},1]}^{\min[\frac{L}{\rho},1]} \mathrm{d}\cos(\theta) \left(1 - \frac{\rho}{L} |\cos(\theta)| \right) \frac{w}{2\rho\delta r_\perp \sin(\theta)} J_1 \left(\frac{2\rho\delta r_\perp \sin(\theta)}{w^2} \right)$$
$$\times \, \mathrm{e}^{-\frac{\delta r_\perp^2 + \rho^2 \sin^2(\theta)}{w^2}} \mathrm{e}^{-\frac{\left(\frac{n_g \rho y}{c} + \delta t - \tau\right)^2}{\tau_\sigma^2}}. \tag{24}$$

Here, J_i are the modified Bessel functions of the first kind. The way the EOS signal is expressed in Eq. (22) as integrals over the space-time distances ρ and τ allows one to use it as a starting point for the discussion of causal and non-causal contributions to the signal in Supplementary Note 2 C below. Before we do so, some approximations to the full expression in Eq. (22) are applied which will simplify the discussion of the result. Note, however, that none of these approximations have been made in order to analyze the experimental data.

B. Simplified expression

In this section we introduce an approximated simplified expression for the EOS signal. We do not use this approximation in order to analyze the experimental data and only introduce it here, such that it can be used in future studies and in order to ease the discussion of the frequency cut-off in the beginning of the next section.

Since the two laser pulses are separated by $\delta r_\perp = 50\mu$m and we expect that most contributions stem from vacuum fluctuations which do not propagate perpendicularly to the propagation direction of the laser pulses (see discussion at the end of Supplementary Note 2 C), we expect to find most contributions for $\rho > 100\mu$m. Thus, we can approximate $k\rho \gg 1$ which allows one to use the retarded limit of the Green's tensor, see Eq. (5). We will further

assume $\rho_x/\rho \ll 1$. Using these approximations the two point correlation function is isotropic, i.e. $S(\mathbf{r}-\mathbf{r}', t-t') \approx S(\rho,\tau)$ such that

$$G^{(1)}_{\mathrm{eo}}(\delta t, \delta r_\perp) = \int_{-\infty}^{\infty} \mathrm{d}\tau \int_0^\infty \mathrm{d}\rho\, \rho^2 S(\rho,\tau) F_1(\rho,\tau) \tag{25}$$

$$= \frac{\hbar}{\epsilon_0 c^2 \pi} \int_{-\infty}^{\infty} \mathrm{d}\tau \int_0^\infty \mathrm{d}\rho\, \rho \int_0^\infty \mathrm{d}\Omega\, \Omega^2 \cos(\Omega\tau) \sin(k\rho) F_1(\rho,\tau). \tag{26}$$

C. Contributions from causal and non-causal quantum vacuum fluctuations

The causal and non-causal contributions to the EOS signal are calculated in this section, using the expressions for the EOS signal in time domain derived in the last two sections. To simplify the discussion we often stick with the approximated result in Eq. (26), but all arguments made using this approximated result are qualitatively also true for the general result in Eq. (22). Note, that the results shown in Fig. 3 of the main text do not assume any of the approximations used in Supplementary Note 2 B.

Equation (26) can be used, in principle, to estimate the contributions to the EOS signal $G^{(1)}_{\mathrm{eo}}(\delta t, \delta r_\perp)$ stemming from non-causal space-time regions by limiting the τ integral to the range $[-\rho n(\Omega)/c, \rho n(\Omega)/c]$. This leads to

$$G^{(1)}_{\mathrm{eo,nc}}(\delta t, \delta r_\perp) = \frac{\hbar}{2\epsilon_0 c^2 \pi^2 w^2 L} \int_0^\infty \mathrm{d}\rho\, \rho \int_{-\min[\frac{L}{\rho},1]}^{\min[\frac{L}{\rho},1]} \mathrm{d}\cos(\theta)$$
$$\times \left(1 - \frac{\rho}{L}|\cos(\theta)|\right) J_0\left(\frac{2\rho\delta r_\perp \sin(\theta)}{w^2}\right) \mathrm{e}^{-\frac{\delta r_\perp^2 + \rho^2 \sin^2(\theta)}{w^2}} \int_0^\infty \mathrm{d}\Omega\, \Omega^2 \mathrm{e}^{-\frac{\Omega^2 \tau_\sigma^2}{4}} \sin(k\rho)$$
$$\times \mathrm{Re}\left\{ \mathrm{e}^{\mathrm{i}\Omega\left(\frac{n_g \rho_y}{c} + \delta t\right)} \left[\mathrm{Erf}\left(\frac{\mathrm{i}\tau_\sigma \Omega}{2} + \frac{n_g \rho_y + n(\Omega)\rho}{c\tau_\sigma}\right) - \mathrm{Erf}\left(\frac{\mathrm{i}\tau_\sigma \Omega}{2} + \frac{n_g \rho_y - n(\Omega)\rho}{c\tau_\sigma}\right) \right] \right\}. \tag{27}$$

The full signal on the other hand is obtained from Eq. (25) by integrating τ from $-\infty$ to ∞ giving

$$G^{(1)}_{\mathrm{eo}}(\delta t, \delta r_\perp) = \frac{\hbar}{\epsilon_0 c^2 \pi^2 w^2 L} \int_0^\infty \mathrm{d}\rho\, \rho \int_{-\min[\frac{L}{\rho},1]}^{\min[\frac{L}{\rho},1]} \mathrm{d}\cos(\theta) \left(1 - \frac{\rho}{L}|\cos(\theta)|\right) J_0\left(\frac{2\rho\delta r_\perp \sin(\theta)}{w^2}\right)$$
$$\times \mathrm{e}^{-\frac{\delta r_\perp^2 + \rho^2 \sin^2(\theta)}{w^2}} \int_0^\infty \mathrm{d}\Omega\, \Omega^2 \mathrm{e}^{-\frac{\Omega^2 \tau_\sigma^2}{4}} \sin(k\rho) \cos\left[\Omega\left(\frac{n_g \rho_y}{c} + \delta t\right)\right]. \tag{28}$$

Using Eqs. (27) and (28), the causal contribution can be obtained via $G^{(1)}_{\text{eo,c}}(\delta t, \delta r_\perp) = G^{(1)}_{\text{eo}}(\delta t, \delta r_\perp) - G^{(1)}_{\text{eo,nc}}(\delta t, \delta r_\perp)$. As can be seen by inserting the asymptotic expansion of the complex error function[6]

$$\text{Erf}(z) \to -\frac{\mathrm{e}^{-z^2}}{z\sqrt{\pi}} \quad \text{for } |z| \to \infty \text{ and } \frac{\pi}{4} < \arg(z) < \frac{3\pi}{4}, \tag{29}$$

the non-causal contribution $G^{(1)}_{eo,nc}$ and consequently also the causal contribution $G^{(1)}_{eo,c}$ *individually* exhibit ultra-violet divergencies, while the full signal $G^{(1)}_{eo}$ remains finite. These divergencies are a common feature in the field of quantum vacuum physics which one e.g. also faces when deriving the Casimir force between two perfectly reflecting mirrors[7]. Here, the divergence can be regularised in a physically meaningful way by recourse to the finite frequency bandwidth of the detection scheme: in the EOS experiment discussed here only vacuum fluctuations up to a certain maximal frequency $\Omega_{\max}$ can be measured. This frequency bandwidth is directly related to and controlled by the pulse duration τ_σ as can be seen from the spectral autocorrelation function $\Gamma^2(\Omega) = \mathrm{e}^{-\Omega^2\tau_\sigma^2/4}$ appearing in Eq. (11) where it effectively limits the frequency bandwidth of the EOS to a finite frequency range. Restricting the analysis of causal and non-causal contributions to the EOS signal to this finite frequency bandwidth thus introduces a natural cut-off of the frequency integral in Eq. (27), i.e. the Ω integral in Eq. (27) is limited to the frequency range $[0\,\text{THz}, \Omega_{\max}]$.

To find a good estimate for $\Omega_{\max}$ we numerically evaluate Eq. (11) only allowing for frequencies up to $\Omega_{\max}$ and compare it to the result without any frequency cut-off. We find that for $\Omega_{\max} \approx 3.5 \times 2\pi$ THz, the ratio of the cut and full signal is given by 96 %, showing that the majority of the signal comes from vacuum fluctuations with frequencies below $3.5 \times 2\pi$ THz. For the experimental parameters used to obtain this result see Supplementary Note 2 D. Note also, that this suppression of the signal for frequencies above $\Omega_{\max} = 3.5 \times 2\pi$ THz is confirmed by calculating the spectral auto-correlation function at $\Omega_{\max}$ which gives $\Gamma^2(\Omega_{\max}) = 3.6\%$ and thus shows a suppression of the signal for $\Omega > \Omega_{\max}$.

Using this frequency cut-off at $\Omega_{\max} \approx 3.5 \times 2\pi$ THz one can numerically evaluate Eqs. (27) and (28). Also using $G^{(1)}_{\text{eo,c}}(\delta t, \delta r_\perp) = G^{(1)}_{\text{eo}}(\delta t, \delta r_\perp) - G^{(1)}_{\text{eo,nc}}(\delta t, \delta r_\perp)$ enables one to find the ratio between the EOS signal at $\delta t = 0$ stemming from causal and non-causal vacuum fluctuations: $G^{(1)}_{\text{eo,c}}(0, \delta r_\perp)/G^{(1)}_{\text{eo,nc}}(0, \delta r_\perp) = -33\,\%$ (again we used the experimental parameters summarized in Supplementary Note 2 D to obtain this result). We find that the majority of the vacuum correlations measured in the experiment are non-causal. Note that a more thorough comparison of the amount of causal and non-causal contributions to the EOS signal, which also accounts for the fact that the contributions of the different frequency modes of the vacuum fluctuations to the EOS signal can have a positive or negative sign, is carried

out at the end of this section, see Eqs. (38) and (39).

Further insight into the space-time structure of the accessed vacuum fluctuations is found by exploiting the correlation measurement performed in the experiment. The spectrum of the measured vacuum fluctuations can be accessed via a Fourier transformation of $G^{(1)}_{\mathrm{eo}}(\delta t, \delta r_\perp)$ as given in Eq. (11) with respect to δt[5,8], i.e. we find

$$G^{(1)}_{\mathrm{eo}}(\Omega, \delta r_\perp) \equiv \frac{1}{2\pi}\int_{-\infty}^{\infty} \mathrm{d}\delta t\, \mathrm{e}^{\mathrm{i}\delta t\Omega} G^{(1)}(\delta t, \delta r_\perp) \tag{30}$$

$$= \frac{1}{2}\int_{V_\mathrm{C}} \mathrm{d}^3 r \int_{V_\mathrm{C}} \mathrm{d}^3 r' F(\mathbf{r}, \mathbf{r}', \Omega) \langle \hat{E}_{\mathrm{vac,x}}(\mathbf{r}, \Omega) \hat{E}^\dagger_{\mathrm{vac,x}}(\mathbf{r}', \Omega)\rangle. \tag{31}$$

Such a relation is also known as the Wiener-Khinchin theorem. Note, that one has to set $\delta t = 0$ in the filter function appearing in Eq. (31). We see in Eq. (31) that obtaining $G^{(1)}(\Omega, \delta r_\perp)$ from a series of correlation measurements with varying δt allows one to access the signal stemming from individual single-frequency mode of the quantum vacuum fluctuations. Such a signal can be directly computed in the time domain by using Eq. (14) and assuming that only a single mode of the vacuum field is present such that $\hat{E}(\mathbf{r}, t) \to \hat{E}_{x,\Omega}(\mathbf{r}, t) \equiv \hat{E}_x(\mathbf{r}, \Omega)\mathrm{e}^{-\mathrm{i}\Omega t}$. This leads to

$$G^{(1)}_{\mathrm{eo}}(\Omega, \delta r_\perp) = \frac{1}{2}\int \mathrm{d}t \int_{V_C} \mathrm{d}^3 r \int \mathrm{d}t' \int_{V_C} \mathrm{d}^3 r' L_1(\mathbf{r}, t) L_2(\mathbf{r}', t') \langle \{\hat{E}_{x,\Omega}(\mathbf{r}, t), \hat{E}_{x,\Omega}(\mathbf{r}', t')\}\rangle. \tag{32}$$

Note, that here again δt has to be set equal to 0. Following the same steps as in Supplementary Note 2 A gives

$$G^{(1)}_{\mathrm{eo}}(\Omega, \delta r_\perp) = \frac{\hbar}{2\epsilon_0 c^2 \pi} \int\limits_{-\infty}^{\infty} \mathrm{d}\tau \int\limits_{0}^{\infty} \mathrm{d}\rho\, \frac{\Omega^2}{k^2\rho} \cos(\Omega\tau)$$
$$\times \Big\{ F_1(\rho, \tau) \left[\sin(k\rho)(k^2\rho^2 - 1) + k\rho\cos(k\rho)\right] - F_2(\rho, \tau) \left[\sin(k\rho)(k^2\rho^2 - 3) + 3k\rho\cos(k\rho)\right] \Big\}. \tag{33}$$

In order to calculate the full contribution $G^{(1)}_{\mathrm{eo}}(\Omega, \delta r_\perp)$ as well as its non-causal component $G^{(1)}_{\mathrm{eo,nc}}(\Omega, \delta r_\perp)$ of each single-frequency mode, the τ integral is either carried out over the full real axis so from $-\infty$ to ∞ or over the non-causal space-time region $[-\rho n(\Omega)/c, \rho n(\Omega)/c]$

only, respectively. This way one obtains

$$G^{(1)}_{\rm eo}(\Omega,\delta r_\perp) = \frac{\hbar}{2\epsilon_0 c^2\pi}\int\limits_0^\infty \mathrm{d}\rho\,\frac{\Omega^2}{k^2\rho}\mathrm{e}^{-\frac{\Omega^2\tau_\sigma^2}{4}}\cos\left(\Omega\frac{n_g\rho_y}{c}\right)$$
$$\times\left\{F_1(\rho)\left[\sin(k\rho)(k^2\rho^2-1)+k\rho\cos(k\rho)\right]-F_2(\rho)\left[\sin(k\rho)(k^2\rho^2-3)+3k\rho\cos(k\rho)\right]\right\}, \quad (34)$$

and

$$G^{(1)}_{\rm eo,nc}(\Omega,\delta r_\perp) = \frac{\hbar}{4\epsilon_0 c^2\pi}\int\limits_0^\infty \mathrm{d}\rho\,\frac{\Omega^2}{k^2\rho}\mathrm{e}^{-\frac{\Omega^2\tau_\sigma^2}{4}}$$
$$\times\,\mathrm{Re}\left\{\mathrm{e}^{\mathrm{i}\Omega\frac{n_g\rho_y}{c}}\left[\mathrm{Erf}\left(\frac{\mathrm{i}\tau_\sigma\Omega}{2}+\frac{n_g\rho_y+n(\Omega)\rho}{c\tau_\sigma}\right)-\mathrm{Erf}\left(\frac{\mathrm{i}\tau_\sigma\Omega}{2}+\frac{n_g\rho_y-n(\Omega)\rho}{c\tau_\sigma}\right)\right]\right\}$$
$$\times\left\{F_1(\rho)\left[\sin(k\rho)(k^2\rho^2-1)+k\rho\cos(k\rho)\right]-F_2(\rho)\left[\sin(k\rho)(k^2\rho^2-3)+3k\rho\cos(k\rho)\right]\right\}. \quad (35)$$

Here, we have defined

$$F_1(\rho) = \frac{1}{2w^2L\pi}\int\limits_{-\min[\frac{L}{\rho},1]}^{\min[\frac{L}{\rho},1]}\mathrm{d}\cos(\theta)\left(1-\frac{\rho}{L}|\cos(\theta)|\right)J_0\left(\frac{2\rho\delta r_\perp\sin(\theta)}{w^2}\right)\mathrm{e}^{-\frac{\delta r_\perp^2+\rho^2\sin^2(\theta)}{w^2}}, \quad (36)$$

$$F_2(\rho) = \frac{1}{2w^2L\pi}\int\limits_{-\min[\frac{L}{\rho},1]}^{\min[\frac{L}{\rho},1]}\mathrm{d}\cos(\theta)\left(1-\frac{\rho}{L}|\cos(\theta)|\right)\frac{w}{2\rho\delta r_\perp\sin(\theta)}J_1\left(\frac{2\rho\delta r_\perp\sin(\theta)}{w^2}\right)\mathrm{e}^{-\frac{\delta r_\perp^2+\rho^2\sin^2(\theta)}{w^2}}. \quad (37)$$

$G^{(1)}_{\rm eo,c}(\Omega,\delta r_\perp)$ can be obtained from Eqs. (34) and (35) via $G^{(1)}_{\rm eo,c}(\Omega,\delta r_\perp) = G^{(1)}_{\rm eo}(\Omega,\delta r_\perp) - G^{(1)}_{\rm eo,nc}(\Omega,\delta r_\perp)$. Equations (34) and (35) have been numerically evaluated for different values of Ω to obtain Fig. 3 of the main text. We find that $G^{(1)}_{\rm eo}(\Omega,\delta r_\perp)$, $G^{(1)}_{\rm eo,c}(\Omega,\delta r_\perp)$ and $G^{(1)}_{\rm eo,nc}(\Omega,\delta r_\perp)$ have positive and negative contributions. This mean to estimate the amount of the EOS signal stemming from causal and non-causal space-time regions we evaluate

$$\frac{\int_0^{\Omega_{\max}}\mathrm{d}\Omega|G^{(1)}_{\rm eo,nc}(\Omega,\delta r_\perp)|}{\int_0^{\Omega_{\max}}\mathrm{d}\Omega|G^{(1)}_{\rm eo,nc}(\Omega,\delta r_\perp)|+|G^{(1)}_{\rm eo,c}(\Omega,\delta r_\perp)|} = 75\,\%, \quad (38)$$
$$\frac{\int_0^{\Omega_{\max}}\mathrm{d}\Omega|G^{(1)}_{\rm eo,c}(\Omega,\delta r_\perp)|}{\int_0^{\Omega_{\max}}\mathrm{d}\Omega|G^{(1)}_{\rm eo,nc}(\Omega,\delta r_\perp)|+|G^{(1)}_{\rm eo,c}(\Omega,\delta r_\perp)|} = 25\,\%. \quad (39)$$

Here, we have used $\Omega_{\max} = 3.5\times 2\pi$ THz as before. Equations (38) and (39) show that 75 % of the measured signal stems from non-causal vacuum fluctuations, whereas causal vacuum fluctuation only amount to 25 %. The existence of these residual causal contributions

might seem surprising, considering that the overlap of the two laser pulses has been almost completely removed. They, however, stem from modes which propagate from one pulse to the other at a very shallow angle compared to the propagation direction of the laser pules (indicated as $\hat{y}$ in Fig. 1 b) of the main text). These modes are also the ones favored by the phase-matching condition, as can be seen from the phase matching factor introduced in Supplementary Ref.[5] which reads $\mathrm{sinc}^2\left[\frac{L}{2}(k_z - n_g\Omega/c)\right] = \mathrm{sinc}^2\left[\frac{L\Omega}{2c}(n(\Omega)\cos(\theta) - n_g)\right]$. Using the measured data for $n(\Omega)$ found in Supplementary Fig. 1a and $n_g = 3.18$, we find that for frequencies larger than $\Omega \approx 1\,\mathrm{THz}$ modes are phase-matched which propagate at an angle $\theta = \arccos[n_g/n(\Omega)] < 0.4\,\mathrm{rad}$ with respect to the propagation direction of the laser pulses and at $\Omega = 2 \times 2\pi\,\mathrm{THz}$ where the signal has its maximum we have $\theta = 0.18\,\mathrm{rad}$.

D. Experimental parameters estimation

Paramount to a correct prediction of the experimental result, is a thorough characterization of the experimental parameters used. Amongst them, the focus should be put in particular on the detection crystal refractive index $n(\Omega)$, the temporal duration of the ultrashort probe pulses τ_p, their transverse Gaussian beam waist w and their distance $\delta r_\perp$. In the following we will provide the methods and techniques used to estimate them.

The refractive index at 4 K of the 1 mm ZnTe used for the theoretical prediction of quantum-vacuum electro-optic correlation $G^{(1)}_{\mathrm{eo}}(\Omega)$ is displayed in Supplementary Fig. 1 a). It has been extrapolated from the detection crystal refractive index at room temperature $n(\Omega, T_r)$ with $T_r = 300\,\mathrm{K}$. In order to do this, we have used the experimental curve expressed in Eq. (40), which describes the dependence in frequency domain of the measured ZnTe refractive index on the crystal temperature T_f

$$n(\Omega, T_f) = n(\Omega, T_r) + 3 \cdot 10^{-4}\Delta T + 2.9 \cdot 10^{-6}\Delta T^2. \tag{40}$$

Here T_r is the room temperature and $\Delta T = T_f - T_r$. The original refractive index at 300 K $n(\Omega, T_r)$ has been measured with a commercial THz Time-Domain Spectroscopy (TDS) system. The relative change in the transmitted THz electric field as a function of frequency has been used to extrapolate the ZnTe parameters according to the procedure described in detail in Supplementary Ref.[9,10]. In Supplementary Fig. 1 a), as a reference, we show also the group refractive index n_g of the near-infrared ultrashort pulses chosen to best reproduce the experimental data $n_g = 3.18$[11].

The temporal extent of the ultrashort pulses τ_p has been measured along the laser optical path via a commercial autocorrelator. From the fitting of the intensity autocorrelation trace,

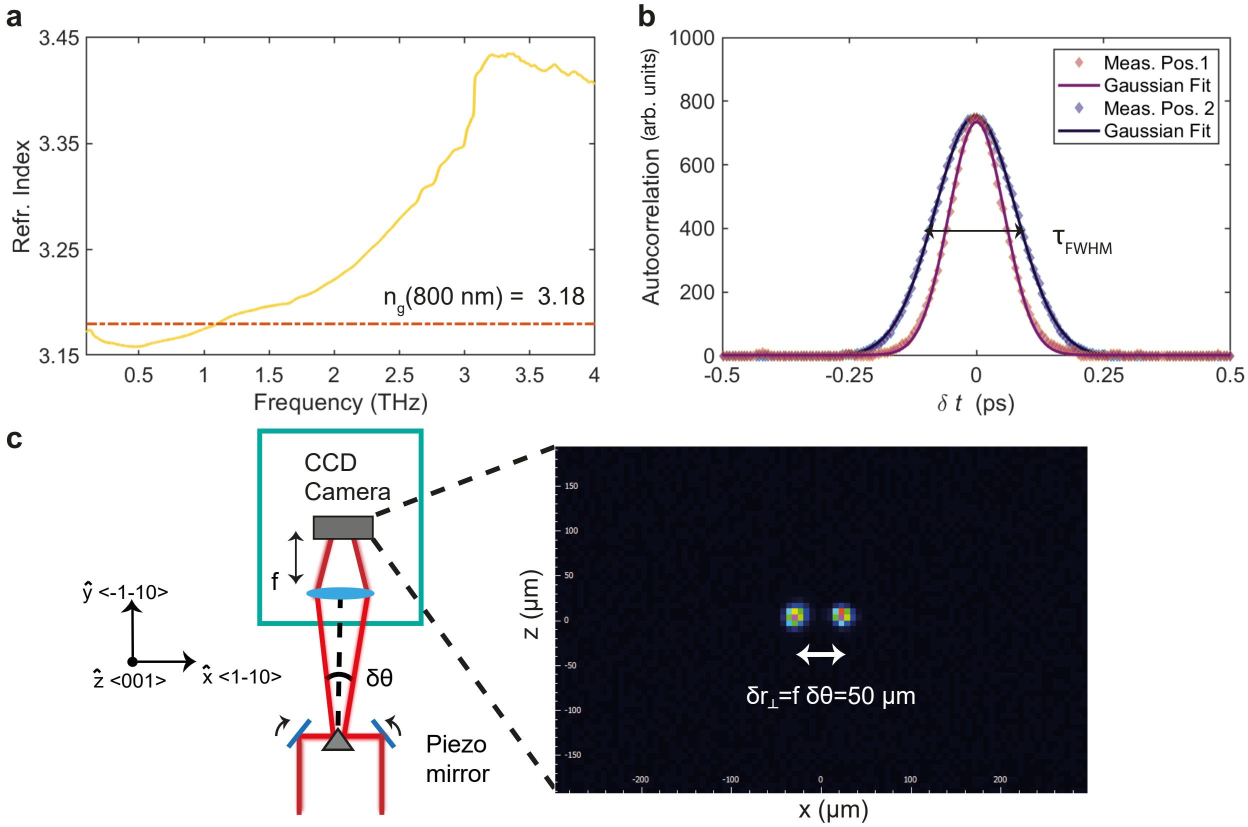

Supplementary Figure 1: **Experimental parameters.** (a) The refractive index of the 1 mm $<110>$-cut ZnTe crystal used for the simulations reported in Fig. 3 of the main text is shown as function of frequency. The value of the group refractive index of ZnTe at 800 nm at 4 K is also reported (dotted-dashed line). The perfect phase matching condition between probing pulses and THz radiation occurs around a frequency of 1 THz. (b) The autocorrelation function of the ultrashort pulse at the laser output (Pos. 1, red) and before being split (Pos.2, blue) is shown. We have retrieved the temporal extent of the pulse from the full-width half maximum (FWHM) τ_{FWHM} of the gaussian fit (solid lines) of autocorrelation functions. The values obtained in this case are equal to $\tau_{\mathrm{p},1} = 80\,\mathrm{fs}$ and $\tau_{p,2} = 133\,\mathrm{fs}$. (c) A sketch of the setup used to determine the near infrared probes spotsize is shown. By projecting the focal plane of the lens onto a near infrared CCD camera, we are able to observe the transverse intensity distribution of the beams (insert). The Gaussian beam waist w of each beam at the focal point has been measured via a built-in software.

one can infer the temporal duration of an ultrashort pulse[12]. In our case, a Gaussian fitting function of the form $f(t) = a \cdot exp(-\frac{2.77 \cdot \delta t^2}{\tau_{FWHM}^2})$ has been chosen. The final temporal extent of the pulse intensity envelope τ_p can be obtained through the relation $\tau_p = 0.707\tau_{\text{FWHM}}$, where τ_{FWHM} is the full width half maximum of the autocorrelation trace.

In Supplementary Fig. 1 b) we present the results of the ultrashort pulse autocorrelation measurement performed at two different locations along the optical path: at the laser output (Pos. 1) and at the entrance of the beam splitter (Pos. 2). The near infrared pulse right at the laser output showed an initial temporal extent of $\tau_{\text{p},1} = 80\,\text{fs}$. Upon propagation along the optical path, the pulse will experience a temporal broadening due to its interaction with the remaining optics. In accordance with Supplementary Ref.[12], the final temporal duration of a Gaussian pulse after propagation through different media can be described via Eq. (41)

$$\tau_p = \tau_{\text{p},1}\sqrt{1 + \frac{(4\ln(2)\sum_i \alpha_i l_i)^2}{\tau_{\text{p},1}^4}}. \tag{41}$$

Here $\tau_{\text{p},1}$ is the duration of the initial Fourier transform limited pulse, l_i represents the thickness of the different optical elements crossed by the near infrared pulse and the coefficient α_i represents their respective group velocity dispersion (GVD). Using Eq. (41), the ultrashort pulse measured at Pos.2 should have a duration of $\tau_{p,2} = 130\,\text{fs}$. The result is in good accord with the experimental measurement, which provides a value of $\tau_{p,2} = 133\,\text{fs}$. The final value of $\tau_p = 195\,\text{fs}$ has been computed using the formula provided in Eq. (41), the lengths of the different optical components present along the optical path $l_1 = 15.9\,\text{mm}$, $l_2 = 25\,\text{mm}$, $l_3 = 10\,\text{mm}$ and the GVD of their respective materials (optical glasses NBK-7 and N-SF1 and calcite,respectively) $\alpha_1 = 44.61\,\text{fs}^2/\text{mm}$, $\alpha_2 = 148.19\,\text{fs}^2/\text{mm}$, and $\alpha_1 = 72\,\text{fs}^2/\text{mm}$ [13].

The experimental setup used to estimate the dimensions of the ultrashort probes beam waist w and their distance $\delta r_\perp$ as a function of the beams relative propagation angle $\delta\theta$ is sketched in Supplementary Fig. 1 c). The focal plane of the lens has been imaged on a infrared CCD camera, which displays the beams intensity profiles on the transverse (x,z) plane (see insert in Supplementary Fig. 1 c)). The beam waist w of the pair of ultrashort pulses has been estimated through a Gaussian fit performed by the camera built-in software.

Their distance $\delta r_\perp$ has been experimentally calibrated as a function of the inclination of the piezo mirrors, which are simmetrically placed around the prism that directs both probes towards the collecting lens inside the dilution fridge. We have defined the transverse distance $\delta r_\perp$ between the probing beams as the relative distance between the position of the maxima of the two intensity distributions in the transverse plane (x,z).

Supplementary Note 3: Data analysis

As described in detail in our previous work (Supplementary material of Supplementary Ref. [8]), the experimental noise affecting our electro-optic correlation setup is of uncorrelated nature. A process of demodulation of the acquired signal at half the repetition rate of the laser $f_{\text{rep}} = 80\,\text{MHz}$ provides an efficient technique for the removal of low frequency drifts (see Methods section of Ref. [8]). In our previous work, we have furthermore proved that our measurement is shot noise limited. The experimental noise can therefore be suppressed by an appropriate choice of the integration time (see Sec. 3 of Supp. Material of Supplementary Ref. [8]).

In order to quantify the experimental uncertainty on the measured quantum vacuum electric field correlation, we also observe electric-field correlations at long time delays $G^{(1)}_{\text{eo}}((\delta t + n \cdot 1/f_{\text{rep}}))$, where n is an integer. At time distances larger than $1/f_{\text{rep}} = 12.5\,\text{ns}$ we expect no vacuum-induced correlation to persist and the resulting signal to arise from noise contributions only.

More specifically, we select as experimental uncertainty per measurement point the standard deviation of the measured electric-field correlation $G^{(1)}_{\text{eo}}(\delta t + 50\,\text{ns})$ acquired with an average distance $\delta r_{\perp} = 50\,\mu\text{m}$ between the sampling beams, shown in Supplementary Fig. 2 a). The experimental value of the uncertainty is equal to $\sigma = 1.05\,\text{V}^2\text{m}^{-2}$.

All experimental measurements reported in this manuscript have been acquired in units of mV^2 as described in Supplementary Note 2 A in Eq. (11). They have been converted in units of V^2m^{-2} using the value of the detection efficiency described in Supplementary Note 2 A in Eq. (16), with an average probe power of $P_t = 740\,\mu\text{W}$ and $P_{\delta t} = 620\,\mu\text{W}$ for the non-delayed and the time delayed probe respectively. These power values have been used to compute the expected shot noise, derived according to Eq. (37) of the supplementary material of Supplementary Ref. [8]. The expected shot noise in units of V^2m^{-2} obtained using the experimental integration time is $\sigma_n = 0.8\,\text{V}^2\text{m}^{-2}$, which is in good agreement with the value of the experimental noise.

In order to prevent the presence of noise-induced artefacts in the frequency domain signal, the experimental data need to be apodized and appropriately filtered. We have chosen to treat the raw data from the vacuum-induced electric-field correlation (in light violet in Fig. 2 in the main text) by applying a Kaiser apodizaition window $\text{K}(\alpha)$. The parameter α is defined as the strength of the windowing function. In order to optimize the strength of the filtering function used, we studied its effect on a noisy signal with a known spectral content. As a reference signal we have used the inverse Fourier transformation of the simulated spectrum obtained according to Eq (3) and shown in Fig. 3 a) (solid black

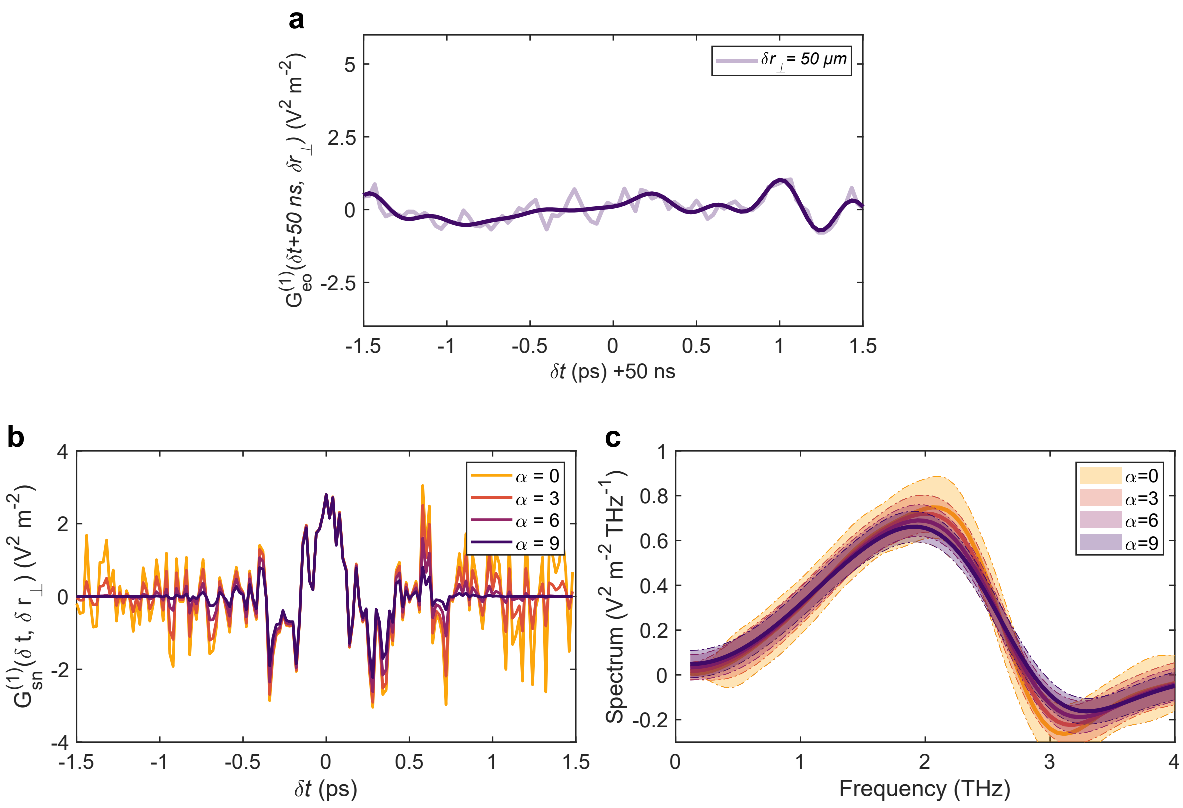

Supplementary Figure 2: **Noise suppression as a function of the different filtering functions strength.** (a) The long time delay correlation trace $G^{(1)}_{\rm eo}(\delta t + 50\,{\rm ns})$ is shown as a function of delay time. It has been measured with the same integration time per point as the experimental timetrace in Fig. 2 (main text) and for the same average beam distance $\delta r_\perp = 50\,\mu{\rm m}$. Its standard deviation is used to estimate the experimental uncertainty and is equal to $\sigma = 1.05\,{\rm V}^2{\rm m}^{-2}$. (b) The time domain signal obtained from the simulated spectrum (Fig. 3 in black in the main text) has been superimposed with random noise with standard deviation σ. The effect of the Kaiser windowing function in time domain is shown for strength values $\alpha = 0, 3, 6, 9$. (c) In frequency domain, we show the effect of the filter on both the simulated spectra (solid lines) and on their relative noise (shaded area) for different values of α. In order to maximise noise suppression and at the same time preserve the spectral bandwidth a value of $\alpha = 6$ has been selected.

line) in the main text. To it we have added a signal obtained from a white noise generator, as it is shown in Supplementary Fig. 2 b). In order to compare the newly obtained noisy signal with the experimental measurement, the noise has been normalized to have the same standard deviation of the experimental uncertainty $\sigma = 1.05\,\mathrm{V^2m^{-2}}$.

The effects of the apodization in frequency domain are reported in Supplementary Fig. 2 c). The Fourier transform of the untreated signal ($\alpha = 0$, yellow solid line in Supplementary Fig. 2 c)) presents a maximum of $0.75\,\mathrm{V^2m^{-2}THz^{-1}}$ at around 2.3 THz and a relative noise amplitude of $\sigma_\alpha = 0.14\,\mathrm{V^2m^{-2}THz^{-1}}$. The latter has been obtained by the statistical distribution of the windowed spectral noise amplitude over 100 different white noise distributions. The use of a windowing function slightly impacts the spectral amplitude of the signal, resulting in a maximum value of $0.71\,\mathrm{V^2m^{-2}THz^{-1}}, 0.68\,\mathrm{V^2m^{-2}THz^{-1}}, 0.66\,\mathrm{V^2m^{-2}THz^{-1}}$ for windowing strengths of $\alpha = 3, 6, 9$ respectively. The relative noise amplitude values at the same time decreases from the original value of $0.14\,\mathrm{V^2m^{-2}THz^{-1}}$ to a value $\sigma_\alpha = 0.11\,\mathrm{V^2m^{-2}THz^{-1}}$,$0.08\,\mathrm{V^2m^{-2}THz^{-1}}$, $0.07\,\mathrm{V^2m^{-2}THz^{-1}}$ for values of $\alpha = 3, 6, 9$ respectively.

A Kaiser window function with a strength of $\alpha = 6$ has been chosen, as it represents a good compromise between a preserved signal spectral amplitude and minimal uncertainty.

Supplementary Note 4: Parasitic low frequency correlations

As shown in Fig. 3 a) in the main text, in the frequency region below 1 THz, a discrepancy between the predicted and measured signal can be observed. The origin of the parasitic low frequency component present in the experimental quantum vacuum-induced electric field correlation could be attributed to either the presence of residual blackbody radiation or to the presence of Kerr-induced third-order nonlinearities.

A. Residual black-body radiation

In order to prevent all external thermal radiation from influencing the experimental result, the ZnTe detection crystal is positioned inside a closed cycle fridge. As sketched in Supplementary Fig. 3 a), the latter consists of a series of thermalized metallic shields with relatively decreasing temperatures. Along the probes optical paths, a series of small apertures have been placed to block thermal radiation and allow the near infrared radiation to propagate through the cryogenic setup.

A system of coupled short focal lenses, which is designed to focus the ultrashort probe

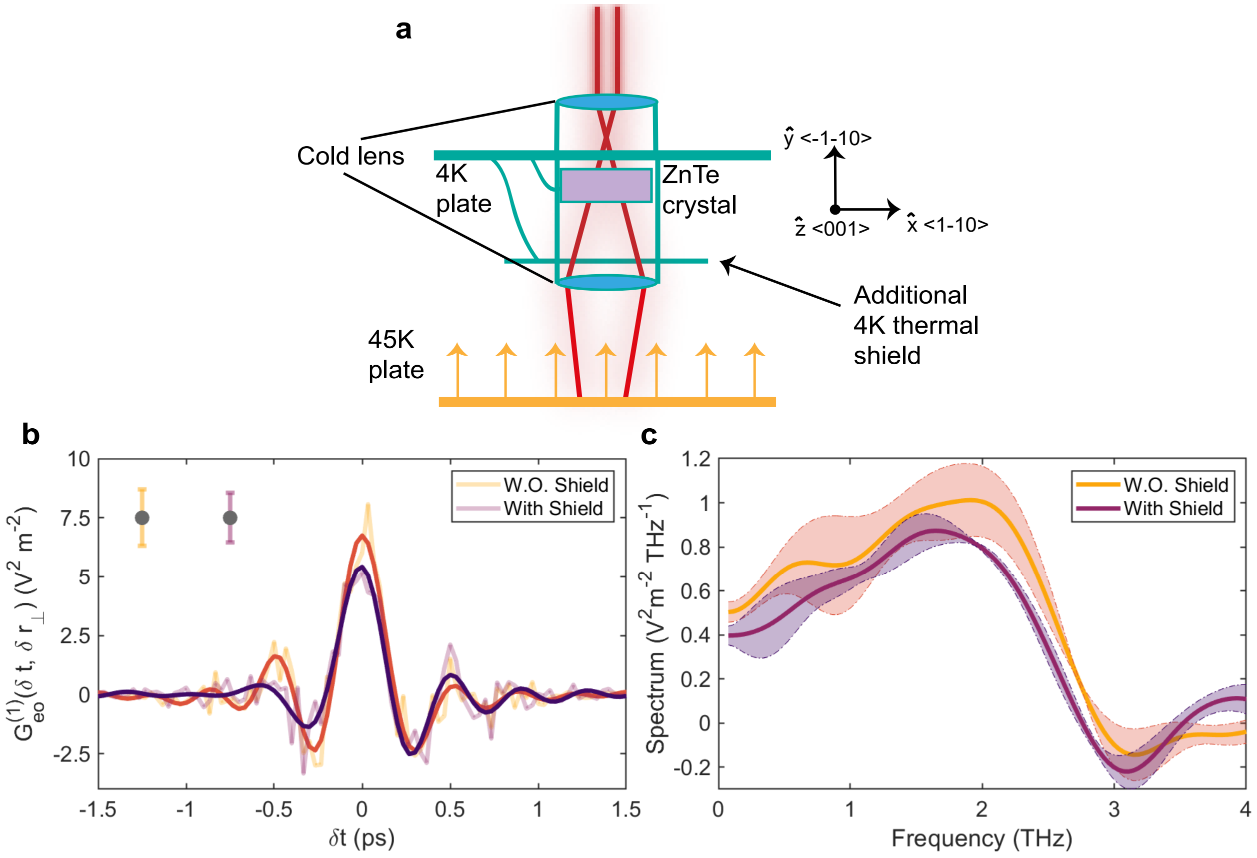

Supplementary Figure 3: **Thermal radiation removal.** (a) The experimental setup in the cryogenic environment consists of a series of concentric shields at low temperatures. The 1mm long ZnTe detection crystal is thermally anchored to a plate with a fixed monitored temperature of 4 K. A pair of cold glass lenses also thermalized at 4 K provide an efficient shield from THz thermal radiation while collecting and focusing the probing near infrared beams in the detection crystal. In this measurement configuration, it is still possible for thermal modes propagating at an angle with respect to the propagation directions of the beams to contribute to the measured experimental signal. In order to prevent it an additional cold shield at 4 K has been added. (b) The comparison between the time traces measured without (yellow faded line) and with (purple faded line) the additional thermal shield is shown. The yellow and purple thick lines represent respectively the experimental measurements performed without and with the additional thermal shield treated with a 3 THz low-pass filter. The uncertainty on the measurements are $\sigma_{W.O.} = 1.2\,\mathrm{V^2m^{-2}}$ and $\sigma_W = 1.05\,\mathrm{V^2m^{-2}}$ for the measurement without and with thermal shield respectively (c). The spectral contents (thick lines) of the two raw measurement reported in (a) are compared. The Fourier transform has been performed using the same filtering described in Supplementary Note 3. The relative spectral amplitude uncertainty is reported as a shaded area.

on the detection crystal and re-collimate them afterwards, is also placed in thermal contact with a metallic shield whose temperature is fixed at 4 K. The cold optical glass of the lenses provides an efficient shielding for thermal radiation in the THz frequency region above 0.5 THz[9], which could impinge on the detection crystal from the adjacent thermal shield at 45 K (orange arrows in Supplementary Fig. 3 a)). The block of 45 K thermal radiation is however efficient only for thermal modes falling inside the solid angle defined by the surface of the cold lens. In order to increase the shielding surface and completely block THz thermally populated modes, we have placed an additional 4K thermal shield between the lens and the detection crystal.

The comparison between the experimental electro-optic correlation $G^{(1)}_{\rm eo}(\delta t, \delta r_\perp)$ obtained in the absence and in the presence of the additional thermal shield is shown in Supplementary Fig. 3 b) . Both measurement have been performed with an average distance between the probing pulses of $\delta r_\perp = 50\,\mu$m. Their experimental uncertainty, estimated as describe in detail in Supplementary Note 3, corresponds to $\sigma_{W.O.} = 1.2\,\mathrm{V^2m^{-2}}$ and $\sigma_{W.} = 1.05\,\mathrm{V^2m^{-2}}$ respectively. As it is shown, the experimental signal measured without and with additional shielding, even if presenting the same features and temporal extension in time domain, displays a variation in amplitude. Increasing the effective solid angle of shielded THz thermally poplated modes reduces the experimental signal from peak value at $\delta t = 0$ of $G^{(1)}_{\rm eo}(\delta t = 0, \delta r_\perp) = 7\,\mathrm{V^2m^{-2}}$ to a value of $G^{(1)}_{\rm eo}(\delta t = 0, \delta r_\perp) = 5\,\mathrm{V^2m^{-2}}$.

In frequency domain (see Supplementary Fig.3 c)), the electric field correlation function $G^{(1)}_{\rm eo}(\delta t, \delta r_\perp)$ reduced amplitude translates into a decrease of the peak amplitude from a value of $G^{(1)}_{\rm eo}(\Omega) = 1\,\mathrm{V^2m^{-2}THz^{-1}}$ centered around $\Omega = 2.2\,\mathrm{THz}$ to a value of $G^{(1)}_{\rm eo}(\Omega) = 0.9\,\mathrm{V^2m^{-2}THz^{-1}}$ around a frequency of $\Omega = 2\,\mathrm{THz}$. We would also like to underline that the bandwidth of the detected signal, dependent on the temperature of the detection crystal only, remains unchanged. The decrease in amplitude of the experimental electro-optic correlation function can be attributed solely to the efficient blocking of thermal radiation.

B. Non-coherent Kerr-induced third order non-linearity

We attribute the discrepancy in the low frequency region between the expected and measured signal shown in Fig. 3 a) of the main text to a non-coherent Kerr-induced third order non-linear effect. Due to the non vanishing value of the third order non-linearity tensor in ZnTe[14], it has already been shown in literature[15] that depending on the relative orientation of the crystallographic axes of the ZnTe crystal with respect to the probes polarization, this effect can become the predominant contribution to the polarization shift experienced by the laser probes. As shown in Supplementary Ref.[15], the effect arises for near infrared

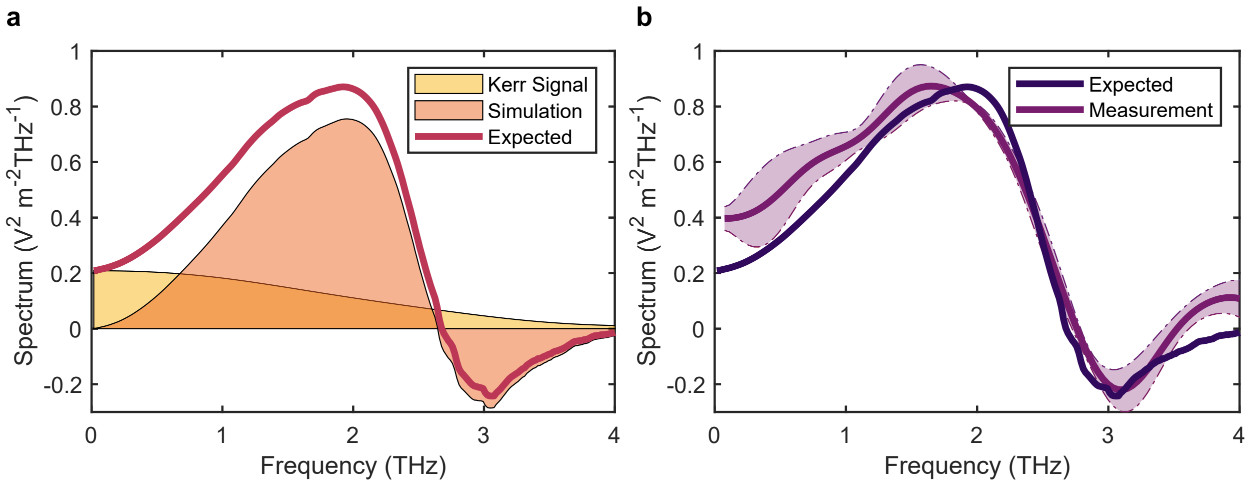

Supplementary Figure 4: **Origin of low frequency components contributions.** a) The scaled Kerr-signal (yellow shaded area) is compared to the quantum vacuum electro-optic signal theoretically predicted (orange shaded area). Their sum will provide the total expected experimental signal (red solid line). b) The measured quantum vacuum correlation (purple solid line) is compared with the total expected signal (violet solid line). The two curves show a good agreement within the experimental uncertainty, especially in the frequency region between $1.75 - 3.25$ THz.

electric fields polarized parallel to the $\hat{z}$ crystallographic axis of the detection ZnTe crystal, which corresponds to the detection configuration implemented in our electro-optic correlation setup.

In analogy with electro-optic detection, electromagnetic vacuum at both THz and at the frequency of the ultrashort probing pulses (near infrared) can be held responsible for the correlated polarization shift experienced from the two laser pulses via a their third order nonlinear interaction. However, due to the direct proportionality of the quantum vacuum modes amplitude on their frequency, contributions arising from electromagnetic vacuum at the same frequency of the ultrashort probing pulses will become predominant.

This Kerr-induced nonlinearity is particularly relevant for probing beams with high peak electric field values. As such, our previous work in Supplementary Ref.[8], which presented a probe peak intensity roughly 100 times smaller than the current measurement configuration, has not been affected by a similar higher order nonlinear effect. Moreover, as described in Supplementary Ref.[14], said Kerr-induced effect can be assumed to be dependent on the spatial and temporal overlap of the intensities of the two interacting beams inside the nonlinear medium. In particular, in frequency domain, Kerr-induced nonlinear electro-optic signal has been shown to be characterized by a high presence of low-frequency contributions.

In order to estimate the third order effect influence for a generic value of the beams transverse separation of $\delta r_\perp$, we have measured the Kerr-induced non coherent electro-optic

signal with $\delta r_\perp = 0\,\mu\text{m}$. This experimental electro-optic signal has been then rescaled according to the normalized value of the tridimensional overlap of the near infrared pulses intensities $\gamma_{ov.}$ inside the nonlinear crystal. The latter has been computed numerically and experimental parameters such as the beam divergence and the non-collinear propagation of the ultrashort probes has been taken into account for the numerical evaluation.

A normalized tridimensional overlap value of $\gamma_{ov.} = 0.001$ has been used to perform the rescaling. The value considered results slightly larger than the one computed for the propagation of the two near infrared probes along a 1 mm ZnTe crystal, an average beam distance of $\delta r_\perp = 50\,\mu\text{m}$ and a Gaussian beam of $w = 10\,\mu\text{m}$. An increased value has been chosen in order to take into account possible aberrations and non-perfect Gaussian behaviour of the experimental ultrashort probing pulses.

The scaled signal obtained for the Kerr-induced measurement with $\delta r_\perp = 50\,\mu\text{m}$ is shown in frequency domain in Supplementary Fig. 4 a). It displays a peak amplitude of $0.2\,\text{V}^2\text{m}^{-2}\text{THz}^{-1}$ and, as expected, it presents mostly contributions from modes with frequency below 1.5 THz. In Supplementary Fig. 4 a) we also report the total expected signal, which can be estimated as the combination of the Kerr-induced non-linear signal and the theoretical prediction for the quantum vacuum correlation function $G^{(1)}_{\text{eo}}(\Omega)$ obtained from Eq. 3 in the main text. The comparison between the expected signal and the experimentally measured one is presented in Supplementary Fig.4 b). As it can be seen, the two spectra not only present the same amplitude of around $0.95\,\text{V}^2\text{m}^{-2}\text{THz}^{-1}$, but also the same bandwidth of about $3.5 \times 2\pi$ THz. The slight mismatch in the peak position of both curves can be attributed to the apodization treatment of the experimental data, as described in detail in Supplementary Note 3.

Supplementary References

(1) Buhmann, S. Y., *Dispersion Forces I: Macroscopic Quantum Electrodynamics and Ground-State Casimir, Casimir–Polder and van der Waals Forces* (Springer, Heidelberg, 2012).

(2) Philbin, T. G.,Canonical quantization of macroscopic electromagnetism *New J. of Phys.* **12**, 123008 (2010).

(3) Scheel, S.,Buhmann, S. Y., Macroscopic quantum electrodynamics - Concepts and applications *Acta Phys. Slovaca* **58**, 5 (2008).

(4) Lindel, F., Bennett, R., Buhmann, S. Y., Theory of polaritonic quantum-vacuum detection *Phys. Rev. A* **102**,041701 (2020).

(5) Lindel, F., Bennett, R., Buhmann, S. Y., Macroscopic quantum electrodynamics approach to nonlinear optics and application to polaritonic quantum-vacuum detection *Phys. Rev. A* **103**, 033705 (2021).

(6) Nijimbere, V., Analytical and asymptotic evaluations of Dawson's integral and related functions in mathematical physics *J. of Appl. Anal.* **25**, 179-188 (2019).

(7) Casimir, H. B. G., On the attraction between two perfectly conducting plates *Proc. K. Ned. Acad Wet.* **51**, 793-795 (1948).

(8) Benea-Chelmus, I-C., Settembrini, F. F., Scalari, G., Faist, J., Electric field correlation measurements on the electromagnetic vacuum state *Nature* **568**, 202-206 (2019).

(9) Naftaly, M., Miles, R. E., Terahertz Time-Domain Spectroscopy for Material Characterization *Proceedings of the IEEE* **95**, 1658-1665 (2007).

(10) Neu, J., Schmuttenmaer, C. A., Tutorial: An introduction to terahertz time domain spectroscopy (THz-TDS) *J. of Appl. Phys.* **124**, 231101 (2018).

(11) Horikoshi, Y., Ebina, A., Takahashi, T., Optical Absorption Due to Acceptor Levels in Undoped ZnTe *Jap. J. of Appl. Phys.* **11**, 992 (1972).

(12) Siegman, A. E., *Lasers* (University Science Books, 1986).

(13) Kartazaev, V., Alfano, R. R., Supercontinuum generated in calcite with chirped femtosecond pulses *Opt. Lett.* **32**, 3293-3295 (2007).

(14) Caumes, J. P., Videau, L., Rouyer, C., Freysz, E., Kerr-Like Nonlinearity Induced via Terahertz Generation and the Electro-Optical Effect in Zinc Blende Crystals, *Phys. Rev. Lett.* **4**, 4-7 (2002).

(15) Tian, Z. et al. Quantitative analysis of Kerr nonlinearity and Kerr-like nonlinearity induced via terahertz generation in ZnTe *App. Phys. Lett.* **4**, 18-11 (2008).
